# The Effects of Salicylic Acid and Its Derivatives on Increasing Pomegranate Fruit Quality and Bioactive Compounds at Harvest and During Storage

**DOI:** 10.3389/fpls.2020.00668

**Published:** 2020-07-01

**Authors:** María E. García-Pastor, Pedro J. Zapata, Salvador Castillo, Domingo Martínez-Romero, Fabián Guillén, Daniel Valero, María Serrano

**Affiliations:** ^1^Department of Agro-Food Technology, University Miguel Hernández, Orihuela, Spain; ^2^Department of Applied Biology, University Miguel Hernández, Orihuela, Spain

**Keywords:** *Punica granatum* L, acetyl salicylic acid, methyl salicylate, anthocyanins, phenolics, ascorbic acid, sugars, organic acids

## Abstract

In the present research two experiments were performed to evaluate the effect of pre-harvest salicylic acid (SA), acetyl salicylic acid (ASA), and methyl salicylate (MeSa), applied as a foliar spray to pomegranate “Mollar de Elche,” on crop yield, fruit quality parameters, and bioactive compounds at harvest and during storage. In the 2017 experiment, trees were treated with SA, ASA, and MeSa at 1, 5, and 10 mM and a higher crop yield (kg tree^–1^ and number of harvested fruit tree^–1^) and quality parameters (firmness, aril color, and individual sugars and organic acids) at harvest were obtained, as well as a higher concentration of phenolics, anthocyanins, and ascorbic acid. The best results were achieved with 10 mM dose of the three assayed compounds, which was chosen for the 2018 experiment, and results for crop yield and fruit quality attributes were confirmed. These quality traits and the concentration of phenolics, anthocyanins, and ascorbic acid were maintained at higher levels in pomegranate fruit from treated trees than in controls during prolonged storage at 10°C. In addition, the effects of salicylate treatments on increasing total and individual anthocyanin concentration in pomegranate arils led to arils with a deeper red color (Graphical Abstract) and, in turn, fruit that would be more appreciated in the international market. This fact, together with the increased crop yield, would contribute to the increased profit of this crop. Thus, pre-harvest treatment with salicylates, and especially SA at 10 mM concentration, could be a safe, natural, and new tool to improve fruit quality and its content on antioxidant compounds with health beneficial effects (namely, ascorbic acid, phenolics, and anthocyanins) at harvest and during storage.

## Graphical Abstract

Experimental design for preharvest salicylate treatments and main results on increasing aril colour.

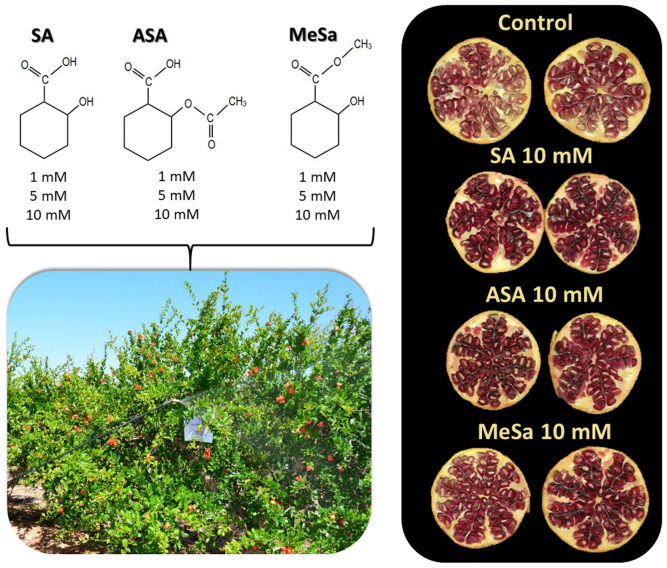

## Introduction

Pomegranate is one of the oldest known edible fruits, associated with ancient civilizations in the Middle East. It was originated in the area nowadays occupied by Iran and Afghanistan and from this area it was spread to India, China, Turkey, Egypt, Tunisia, Morocco, and Spain (Pareek et al., 2015). Spain is the major producer and exporting country of pomegranate fruit in the European Union, with a production area of 3,567 ha and a production of 65,165 t in 2017 (MAGRAMA, 2017). The edible portion of pomegranate are the arils, which consist of around 80% juice and 20% seed (Cristofori et al., 2011) and the fruit can be consumed as fresh fruit or used to prepare juices, canned beverages, jellies, jams, and flavorings and colorings for drinks (Valero et al., 2014).

Pomegranate fruit quality depends largely on fruit size, skin color, and absence of visual defects, such as sunburn, growth cracks, cuts, bruises, and decay, as well as on aril color, sugar and acid content, and the presence of small and soft seeds. In Spain, “Mollar de Elche” is the most cultivated pomegranate cultivar which is very appreciated by consumers due to its high concentration of sugars, low acidity, and its barely discernible seeds, since they are very small and soft and can be easily eaten (Nuncio-Jáuregui et al., 2014). On the other hand, pomegranate has been used in the folk medicine of many countries from ancient times, and its beneficial effects against several diseases, such as atherosclerosis, inflammatory and infective-mediated diseases, Alzheimer, diabetes, infarct brain ischemia, and several types of cancer, have been reported recently and attributed to its phenolic compounds and anthocyanins (Faria and Calhau, 2011; Ismail et al., 2012; Asgary et al., 2017; Panth et al., 2017). Nevertheless, the phytochemical composition of pomegranate fruit is affected by several factors, such as genotype, area of cultivation, environmental conditions, and agronomic management, among others. Thus, six different anthocyanins, namely, cyanidin-3,5-diglucoside, pelargonidin-3,5-diglucoside, delphinidin-3,5-diglucoside, cyanidin-3-glucoside, pelargonidin-3-glucoside, and delphinidin-3-glucoside, were identified in a wide range of Italian pomegranate cultivars, although their concentrations were different depending on cultivars, as well as other phenolic compounds (Fanali et al., 2016; Russo et al., 2018). Great variations on total and individual phenolic and anthocyanin concentrations were also found by Zaouay et al. (2012) in thirteen Tunisian cultivars. However, “Mollar de Elche” cultivar does not have a high anthocyanin content as compared with other worldwide-known cultivars such as “Wonderful” and, in turn, its arils are only slightly red-colored and the skin has a cream-pink color (Li et al., 2015; Pareek et al., 2015; Cano-Lamadrid et al., 2018). This fact makes it difficult for this cultivar to reach international markets. Thus, treatments to increase the coloration of the skin and arils of “Mollar de Elche” pomegranate would lead to its increased commercialization in international markets as well as its antioxidant properties and health beneficial effects. In this sense, it has been reported that water restrictions applied in summer (during the linear phase of fruit growth) led to an increase in aril anthocyanin content (Bartual et al., 2015), as well as treatment with methyl jasmonate during on-tree pomegranate fruit development (García-Pastor et al., 2020).

Salicylic acid (SA) and its derivatives, acetyl salicylic acid (ASA) and methyl salicylate (MeSa), are plant hormones that play important roles in a wide range of physiological processes, from seed germination to flowering and fruit ripening, although the most studied roles have been their effects on inducing plant defense systems against different biotic and abiotic stresses (Tiwari et al., 2017; Koo et al., 2020). It has been reported that postharvest treatments with salicylates reduce decay and chilling injury in a wide range of fruits, and improve other quality properties, such as appearance, texture, and nutritional compounds (Asghari and Aghdam, 2010; Glowacz and Ree, 2015). Thus, SA, ASA, or MeSa applied as postharvest treatments resulted in higher quality attributes in apricots (Wang et al., 2015) and increased the content on anthocyanins and other bioactive compounds in blood oranges (Habibi et al., 2020), sweet cherries (Valero et al., 2011), kiwifruit (Zhang et al., 2003), mango (Ding et al., 2007), sugar apples (Mo et al., 2008), and peaches (Tareen et al., 2012), with additional effects on delaying the postharvest ripening process. On the other hand, the application of these salicylates during fruit development on trees has been reported to improve fruit quality parameters at harvest. Thus, SA treatment at 0.1 and 0.2 mM of vine (at veraison stage) increased anthocyanin content in berries (Oraei et al., 2019) and SA, ASA, or MeSa treatments (0.5, 1, and 2 mM) applied at three key points of fruit development enhanced sugars, organic acids, and antioxidant compounds in plums at harvest and after storage (Martínez-Esplá et al., 2017, 2018) as well as in sweet cherries (Giménez et al., 2014, 2015, 2017; Valverde et al., 2015). In these previous papers, a delay on the postharvest ripening process was also observed, which was attributed to the increased concentration of antioxidant compounds and the activity of antioxidant enzymes.

Specifically, in pomegranate SA, ASA, and MeSa postharvest treatments reduced chilling injury (CI) and maintained fruit quality and higher levels of total antioxidant compounds, such as anthocyanins, phenolics, or ascorbic acid (Sayyari et al., 2009, 2011a,b). Accordingly, postharvest 2 mM SA treatment in combination with a controlled atmosphere storage delayed quality losses and extended the storage life of “Hicazna” pomegranates (Koyuncu et al., 2019). In addition, 0.25 mM ASA treatment of pomegranate arils reduced browning and maintained higher concentrations of phenolics and anthocyanins during storage (Dokhanieh et al., 2016). As a preharvest treatment, just one previous paper is available in which 1 mM SA treatment reduced bacterial blight, a devastating pomegranate disease caused by *Xanthomonas axonopodis pv. Punicae* (Lalithya et al., 2017). However, there are no previous reports regarding the effect of pre-harvest treatments of pomegranate trees with SA, ASA, or MeSa on fruit growth and ripening, as well as on fruit quality attributes at harvest, which was the main goal of the present research. In addition, it was hypothesized that an increase on anthocyanin content would occur as a consequence of these treatments, according to results on other fruit species obtained in previous reports, as commented above.

## Materials and Methods

### Plant Material and Experimental Design

The experiments (2017 and 2018) were performed in a commercial orchard of ‘Mollar de Elche’ pomegranate trees (10,11 years-old), planted at 6 × 5 m, located in Elche, south of Alicante, Spain (UTMX: 694006.000 UTMY: 4234860.000). Climatic conditions in the crop field were: a semi-arid Mediterranean climate, with mean annual temperatures of 19.28 and 18.97°C for 2017 and 2018, respectively; maximum temperatures in summer, from June to September, of 31.62 and 31.42 for 2017 and 2018, respectively; and accumulated rainfall of 238.32 and 270.19 mm for 2017 and 2018, respectively^1^. Soil was composed of sand, silt, and clay at 30, 34, and 36%, respectively, and had a pH of 7.8. Irrigation was carried out by using a drip irrigation system with eight emitters per tree, each delivering 4 L h^–1^ as follows. April: two watering cycles of 1 h per week; May, June, July, August, and September: two watering cycles of 2 h per week; and October: one watering cycle of 1 h. The irrigation water used had an electrical conductivity ca. 3 dS m^–1^. Fertilization was applied in the irrigation system at 160/80/160 kg ha^–1^ nitrogen/phosphorus/potassium (N/P/K) ratio. Pruning and thinning were carried out during the experiments according to standard cultural practices for pomegranate. In 2017 experiment, three blocks or replicates (of three trees each) were selected, totally at random, for treatments: SA, ASA, and MeSA at 1, 5, and 10 mM and control. Treatments were performed by foliar spray application of 3 L of freshly prepared SA, ASA, or MeSa (Sigma Aldrich, Madrid) solutions, containing 1 mL L^–1^ Tween-20 (a polyoxyethylene sorbitol ester, acting as a non-ionic detergent). Control trees were sprayed with distilled water containing 1 mL L^–1^ Tween-20. Dates of treatments were set by taking into account the harvest dates of this cultivar in similar growing conditions in previous seasons, so that the first treatment was applied before the skin color changes and the last one 4 days before the first harvest, according to previous reports (García-Pastor et al., 2020). Dates of treatments in 2017 were: 3rd July, 2nd August, 1st September, and 2nd October. In the 2018 experiment, treatments were SA, ASA, or MeSa at 10 mM concentration and control, which were applied as in the 2017 experiment but instead using five trees for each of the three blocks or replicates. Dates of treatments were: 10th July, 10th August, 11th September, and 11th October. For both years, each block or replicate for each treatment was set in a row, leaving an untreated tree between each block and an untreated row between each treated row in order to avoid treatment cross effects. In addition, at least one tree without treatment was left in each row to avoid edge effect.

Fruits were harvested according to commercial criteria based on fruit size (8.5–9.0 cm of diameter and weight of 325–350 g), skin color (cream-light pink), and total soluble solids (TSS) content characteristic of this cultivar (more than 15°Brix). In both years, fruits were picked on two dates, separated by 20 days, due to the heterogeneous fruit on-tree ripening process. On both harvest dates, the yield (kg tree^–1^ and number of fruits tree^–1^) were determined. In the 2017 experiment, five fruits from the first picking, homogenous in size and color and without visual defects, were selected from each replicate and treatment and immediately transferred to the laboratory for analytical determinations. In the 2018 experiment, four lots of five fruits also homogenous in size and color and without visual defects were chosen at random for each replicate and treatment, immediately transferred to the laboratory, and stored for 0 (day 0), 30, 60, and 90 days at 10°C and at a relative humidity of 85–90%. For each sampling date during storage, one lot was taken at random for each replicate and treatment.

### Fruit Yield, Respiration Rate and Qualitative Traits

Yield, expressed as kg tree^–1^ and number of fruit tree^–1^, was measured at two harvest dates and growing seasons. Then, fruit mass (g) and the percentage of fruits harvested at the first picking were calculated. Results were expressed as the mean ± SE. The weight of each pomegranate lot was measured at day 0 and after each storage period, and weight loss was expressed as a percentage with respect to initial weight. To quantify respiration rate, each fruit lot was hermetically sealed in a 3 L jar for 30 min. After that, 1 mL from the holder atmosphere was withdrawn with a syringe and injected into a Shimadzu TM 14A gas chromatograph (Kyoto, Japan) equipped with a thermal conductivity detector under the chromatographic conditions previously described (Sayyari et al., 2011b). Respiration rate was expressed as g of CO_2_ released by kg^–1^ s^–1^.

Fruit firmness was measured individually in each of the five fruits of each replicate by using a TX-XT2i Texture Analyzer (Stable Mycrosystems, Godalming, United Kingdom) which applied a force to achieve a 3% deformation of the fruit diameter. Results were expressed as the relation between the applied force and the traveled distance (kN m^–1^) and are the mean ± SE.

Skin and aril color were measured by digital image analysis. Photographs of the pomegranates were captured using a digital camera (Nikon D3400) in a light box with black background. The setup conditions of the camera were as follows: light provided by two LEDs of color temperature of 5600 K, flash speed of 1/5 s, ISO-200, focal opening (f) 20, and length 35 mm. For skin color measure, one image of the front and another of the back side of the five fruits of each of the three replicates for each treatment were captured, saved as JPEG file, and analyzed by using the software ImageJ v1.52a (NIH Image, National Institutes of Health, Bethesda, United States). The CIELab model was used to calculate Hue angle (arctg b^∗^/a^∗^) according to García et al. (2017). Pomegranate fruits were cut by the equatorial plane and aril color was measured by taking photographs of the cut surface as indicated above. After that, arils of the five pomegranates of each replicate were combined to obtain a homogeneous sample for each replicate in which the following parameters were measured. Titratable acidity (TA) was determined in duplicate in each sample, by using 1 mL of diluted juice (in 25 mL distilled H_2_O), obtained from 30 g of pomegranate arils, which was automatically titrated (785 DMP Titrino, Metrohm) with 0.1 N NaOH up to pH 8.1, and the results were expressed as g malic acid equivalent kg^–1^ in fresh weight basis. Total soluble solids (TSS) were measured in duplicate in the same juice by using a digital refractometer (Atago PR-101, Atago Co., Ltd., Tokyo, Japan) at 20°C and expressed as g kg^–1^ in fresh weight basis. After that, the aril samples were stored at −25°C until the following determinations were performed.

### Total Phenolics, Total Anthocyanin, and Total Antioxidant Activity Quantification

To extract phenolic compounds, 5 g of arils were homogenized with 10 mL of water: methanol (2:8, v/v) containing 2 mM NaF by using a homogenizer (Ultraturrax, T18 basic, IKA, Berlin, Germany) for 30 s. The extracts were centrifuged at 10,000 *g* for 10 min at 4°C and the supernatant was used to quantify total phenolics (in duplicate in each extract) by using the Folin-Ciocalteu reagent as previously described by Sayyari et al. (2011a). The results were expressed as g gallic acid equivalent (GAE) kg^–1^ and are the mean ± SE of three replicates. For anthocyanin extraction, 5 g of arils were homogenized as above in 15 mL of methanol: formic acid: water (79:1:20, v/v/v) and then centrifuged at 10,000 *g* for 10 min at 4°C. The absorbance at 520 nm was measured in the supernatant (in duplicate for each sample) and total anthocyanin content (TAC) was expressed as g kg^–1^ of cyanidin 3-*O*-glucoside equivalents (Cyn 3-gluc, molar absorption coefficient of 23,900 L cm^–1^ mol^–1^ and molecular weight of 449.2 g mol^–1^). To measure total antioxidant activity (TAA), 5 g of arils were homogenized in 10 mL of 50 mM phosphate buffer pH = 7.8 and 5 mL of ethyl acetate as indicated above. The homogenate was centrifuged at 10,000 *g* for 15 min at 4°C and the upper and lower fractions were used to quantify lipophilic (L-TAA) and hydrophilic total antioxidant activity (H-TAA), respectively. H-TAA and L-TAA were determined in duplicate in each extract as previously described (Sayyari et al., 2011a), in a reaction mixture containing 2,20-azino-bis-(3-ethylbenzothiazoline-6-sulfonic acid) diammonium salt (ABTS), horseradish peroxidase enzyme, and its oxidant substrate (hydrogen peroxide), in which ABTS^+^ radicals are generated and monitored at 730 nm. The decrease in absorbance after adding the pomegranate extract was proportional to TAA of the sample which was calculated by using a calibration curve made with Trolox [(R)-(+)-6-hydroxy- 2, 5, 7, 8-tetramethyl-croman-2-carboxylic acid] (0–20 nmol) from Sigma Aldrich (Madrid, Spain), and results are expressed as g of Trolox Equivalent (TE) kg^–1^ and are the mean ± SE of three replicates.

### Individual Anthocyanin Quantification

The extracts obtained for total anthocyanin quantification described previously were filtered through a 0.45 μm PVDF filter (Millex HV13, Millipore, Bedford, MA, United States) and then individual anthocyanins were identified by liquid chromatography coupled to mass spectrometry (HPLC-DAD-ESI/MSn) by using an Agilent HPLC1100 series machine equipped with a photodiode array detector and a mass detector in series (Agilent Technologies, Waldbronn, Germany), as previously reported (Martínez-Esplá et al., 2014). To quantify individual anthocyanins, two samples of each extract were injected into a HPLC system (Agilent HPLC 1200 Infinity series) working with the chromatographic conditions previously reported (Martínez-Esplá et al., 2014). Chromatograms were recorded at 520 nm and quantification was performed by using calibration curves carried out with cyanidin 3-*O*-glucoside (Cyn 3-gluc), cyanidin 3,5-*O*-di-glucoside (Cyn 3,5-di-gluc), pelargonidin 3-*O*-glucoside (Plg 3-gluc), and pelargonidin 3,5-*O*-di-glucoside (Plg 3,5-di-gluc) (Sigma-Aldrich, Germany). Delphinidin 3-*O*-glucoside (Dlp 3-gluc) and delphinidin 3,5-*O*-di-glucoside (Dlp 3,5-di-gluc) were quantified as Cyn 3-gluc equivalents. Results were expressed as mg kg^–1^ fresh weight (mean ± SE of three replicates).

### Ascorbic Acid (AA), Dehydroascorbic Acid (DHA), and Total Vitamin C Quantification

Ascorbic (AA) and dehydroascorbic (DHA) acids were measured according to Peña-Estévez et al. (2016). Briefly, 5 g of frozen arils were homogenized with 5 mL of methanol: water (5:95) containing 0.1 mM citric acid, 0.05 mM ethylenediamine tetraacetic acid disodium salt, and 4 mM NaF for 30 s on an Ultraturrax, T18 basic, IKA, Berlin, Germany. Then, the extract was filtered through a four-layer cheesecloth, the pH was adjusted to 2.35–2.40 with 2 N ClH, and centrifuged at 10,000 *g* for 15 min at 4°C. The supernatant was purified through a methanol-activated C18 cartridge (Sep-Pak cartridges C18, Waters, Dublin, Ireland) and filtered through a 0.45 μm PFTE filter. For DHA derivatization, 750 μL of extract were mixed with 250 μL of 7.7 M 1,2-phenylenediamine in an HPLC amber vial. The mixture was allowed to react for 37 min and then 20 μL were injected onto a Luna (250 mm × 4.6 mm, 5 μm particle size) C18 column (Phenomenex, Macclesfield, United Kingdom) with a C18 security guard (4.0 mm × 3.0 mm) cartridge system (Phenomenex, Macclesfield, United Kingdom) using a HPLC system (Agilent HPLC 1200 Infinity series). The mobile phase was 50 mM KH_2_PO_4_ containing 5 mM hexadecyl trimethyl ammonium bromide and 5% methanol (pH 4.59) with isocratic flow of 1 mL min^–1^. Absorbance was recorded at 261 nm for AA (Rt = 9.4 min) and at 348 nm for DHA (Rt = 4.5 min) and they were quantified by comparison with AA and DHA standards areas (Sigma-Aldrich, Germany). The results (mean ± SE) were expressed as g kg^–1^ fresh weight.

### Individual Sugars and Organic Acids Content

To measure sugars and organic acids, 5 g of the aril sample of each replicate were extracted with 5 mL of 0.5% phosphoric acid and the supernatant was filtered through 0.45 μm Millipore filter and injected in duplicate into a HPLC system (Hewlett-Packard HPLC series 1100). The elution system consisted of 0.1% phosphoric acid running isocratically at 0.5 mL min^–1^ through a Supelco column (Supelcogel C-610H, 30 cm 7.8 mm, Supelco Park, Bellefonte, United States). Organic acids were detected by absorbance at 210 nm and sugars by refractive index detector and quantified by using standard curves of pure sugars and organic acids (Sigma-Aldrich, Germany). Results were expressed as g kg^–1^ fresh weight and are the mean ± SE of three replicates.

### Statistical Analysis

Results are expressed as mean ± SE of three replicates. Data for the analytical determinations were subjected to analysis of variance (ANOVA) being sources of variation treatment for the 2017 experiment and storage time and treatment for the 2018 experiment. Mean comparisons were performed using HSD Tukey’s test to examine if differences between control and treated fruit were significant at *P* < 0.05. All analyses were performed with SPSS software package v. 17.0 for Windows.

## Results

### Crop Yield

SA treatments of pomegranate trees during the development of pomegranate fruit increased crop yield in a dose-dependent way, the effect being significant (*P* < 0.05) with 5 and 10 mM. Thus, the yield in the 2017 experiment, expressed as kg tree^–1^, was 37.75 ± 3.28 in control trees and 51.08 ± 6.52 in those treated with 10 mM SA. Yield was also increased by MeSa treatments although no significant differences were observed among 1, 5, and 10 mM doses. These increases were due to the higher number of fruits harvested from each tree, while fruit mass was not affected by treatment. However, ASA treatments did not have a significant effect (*P* < 0.05) on crop yield, neither on kg tree^–1^ nor on number of fruit tree^–1^ (Table 1). These results were confirmed in the 2018 experiment, in which 10 mM dose was applied for SA, ASA, and MeSa treatments (Table 1). The percentage of fruits that were harvested in the first picking date was 55.32 ± 3.18% in control fruits which was increased, in a dependent-concentration manner, by SA and ASA treatments, with ca 90% of total fruit being harvested at the first picking date on SA and ASA 10 mM treated fruits. However, the percentage of fruits picked at the first picking date on trees treated with MeSa, either at 1, 5, or 10 mM concentration, was similar to those of non-treated trees (Table 1).

**TABLE 1:** Effects of preharvest salicylic acid (SA at 1, 5, and 10 mM), acetyl salicylic acid (ASA at 1, 5, and 10 mM), and methyl salicylate (MeSa at 1, 5, and 10 mM) treatments on pomegranate crop yield (kg tree^–1^ and number of fruit tree^–1^), fruit mass, and% of fruits harvested in the first picking date in the 2017 and 2018 experiments.

	kg tree^–1^	Fruits tree^–1^	Fruit mass (g)	% first pick
**2017 experiment**				
Control	37.54 ± 3.28 a	110.96 ± 7.17 a	338.3 ± 9.54 a	55.32 ± 3.18 a
SA 1	41.45 ± 3.14 ab	123.8 ± 5.2 ab	334.8 ± 9.27 a	49.61 ± 2.65 a
SA 5	45.59 ± 4.29 bc	135.6 ± 6.1 bc	336.2 ± 7.48 a	81.87 ± 2.77 c
SA 10	51.08 ± 3.52 c	141.8 ± 5.7 c	352.9 ± 11.8 a	88.48 ± 3.18 d
ASA 1	42.62 ± 3.56 ab	127.8 ± 4.6 b	333.5 ± 12.8 a	63.17 ± 1.11 b
ASA 5	39.70 ± 3.58 a	117.21 ± 6.6 a	338.7 ± 10.3 a	84.67 ± 4.14 cd
ASA 10	42.39 ± 2.08 ab	124.9 ± 3.74 ab	339.2 ± 12.1 a	88.84 ± 3.64 d
MeSa 1	50.90 ± 3.78 c	142.0 ± 6.4 c	358.0 ± 9.89 a	54.63 ± 5.51 a
MeSa 5	50.43 ± 2.74 c	152.6 ± 9.57 c	330.5 ± 15.3 a	50.28 ± 4.70 a
MeSa 10	50.39 ± 2.66 c	151.0 ± 7.8 c	333.8 ± 16.2 a	49.69 ± 4.61 a
**2018 experiment**				
Control	43.42 ± 4.06 a	119.6 ± 9.28 a	362.9 ± 11.1 a	55.21 ± 4.22 a
SA 10	51.39 ± 2.90 b	145.7 ± 8.82 b	352.8 ± 9.81 a	65.67 ± 3.70 b
ASA 10	41.41 ± 3.99 a	118.7 ± 8.03 a	348.9 ± 13.3 a	64.59 ± 3.80 b
MeSa 10	55.96 ± 4.70 b	150.0 ± 13.6 b	372.9 ± 7.75 a	47.91 ± 3.96 a

*Within a column, different letters show significant differences (*P* < 0.05) among treatments.*

### Fruit Quality Parameters and Bioactive Compounds at Harvest

**FIGURE 1:**
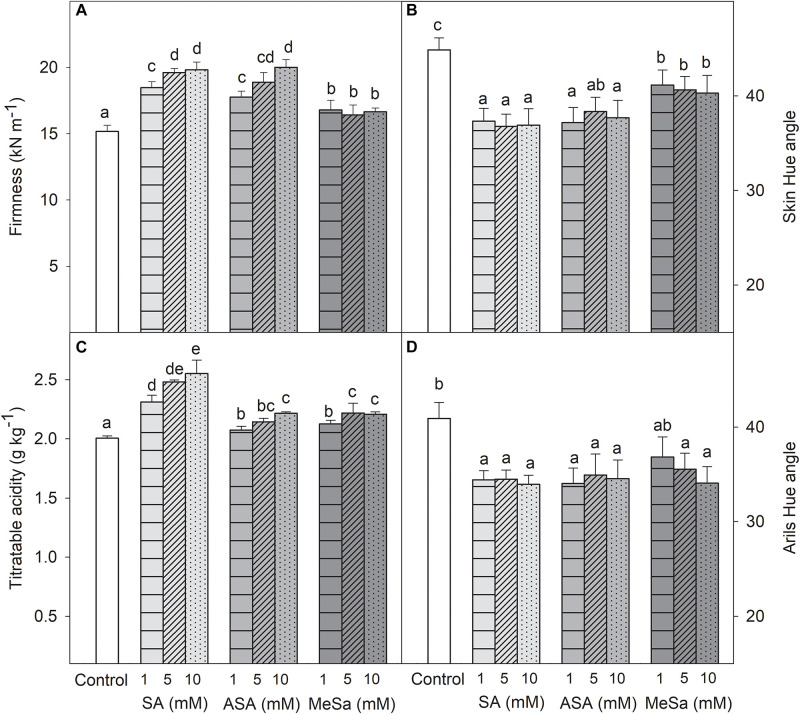
Fruit firmness **(A)**, skin color (Hue angle) **(B)**, titratable acidity **(C)**, and arils color (Hue angle) **(D)** in pomegranate from control and salicylic acid (SA), acetyl salicylic acid (ASA), and methyl salicylate (MeSa) treated fruits at harvest, in the 2017 experiment. Data are the mean ± SE. Different letters show significant differences (*P* < 0.05) among treatments.

Fruit quality parameters, such as firmness, TA, and skin and aril color, at harvest, were improved by all salicylate treatments in the 2017 experiment. Thus, fruit firmness and TA were significantly higher (*P* < 0.05) in all SA, ASA, and MeSa treated fruits than in controls, the effects being, in general, dose-dependent and higher for SA and ASA treatments than for MeSa (Figures 1A,C). On the contrary, Hue angle in skin and arils was significantly (*P* < 0.05) lower in all treated fruits with respect to control ones (Figures 1B,D), which show a deep red color of both skin and arils as a consequence of treatments, although no significant differences were observed among treatments. TSS at harvest were 158.5 ± 1.0 g kg^–1^ in arils control fruits and significantly (*P* < 0.05) higher in 10 mM SA, ASA, and MeSa treated ones, 173.8 ± 0.7, 168.9 ± 0.9, and 169.7 ± 1.2 g kg^–1^, respectively, while no significant effects were observed for salicylate treatments at 1 and 5 mM (data not shown).

**FIGURE 2:**
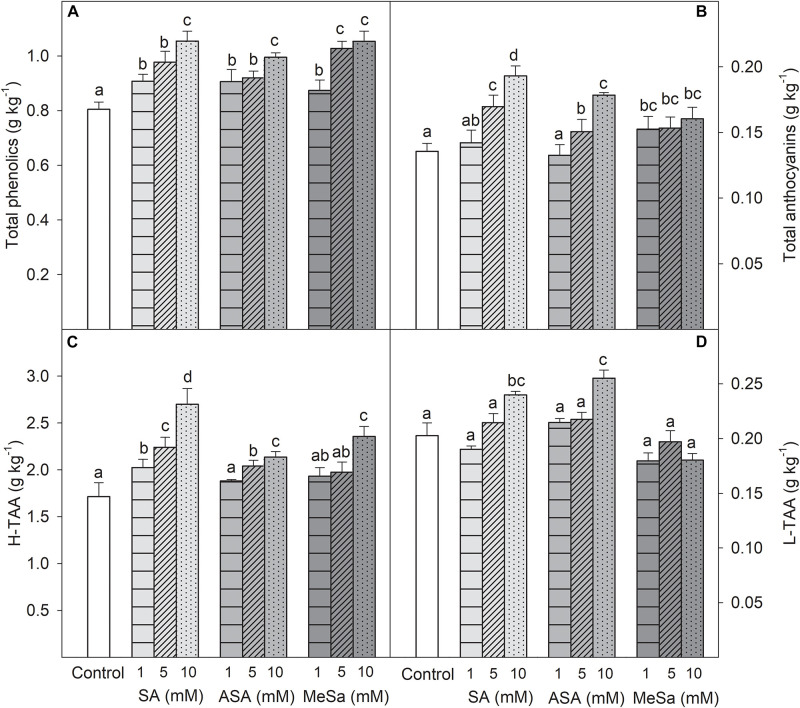
Total phenolic concentration **(A)**, total anthocyanin concentration **(B)**, total hydrophilic antioxidant activity (H-TAA) **(C)**, and total lipophilic antioxidant activity (L-TAA) **(D)** in arils of pomegranate from control and salicylic acid (SA), acetyl salicylic acid (ASA), and methyl salicylate (MeSa) treated fruits at harvest, in the 2017 experiment. Data are the mean ± SE. Different letters show significant differences (*P* < 0.05) among treatments.

In 2017, SA, ASA, and MeSa treatments also induced a significant (*P* < 0.05) increase in total phenolics and anthocyanin concentrations in pomegranate arils, which was higher as increased the applied dose (Figures 2A,B). A similar trend was observed for H-TAA, the highest increase being observed on arils of 10 mM SA treated fruits (Figure 2C). However, for L-TAA the effects of salicylate treatments were not so evident, since it was significantly (*P* < 0.05) increased only by SA and ASA 10 mM treatments (Figure 2D). Taking into account the results obtained in the 2017 experiment, SA, ASA, and MeSa treatments were applied at 10 mM in 2018 and fruits were used for the storage experiment.

### Evolution of Pomegranate Quality Parameters and Respiration Rate During Storage

Weight loss increased along storage time, reaching final values of 9.42 ± 0.43% in control fruits and significantly (*P* < 0.05) lower 5.90 ± 0.49, 7.02 ± 0.36, and 7.88 ± 0.58% in fruits from SA, ASA, and MeSa treated ones, respectively (Supplementary Figure S1A). Respiration rate at harvest was 86.36 ± 1.14 g kg^–1^ s^–1^ in control fruit and this was significantly (*P* < 0.05) higher than those from SA treated trees (76.30 ± 2.52 g kg^–1^ s^–1^).However, no significant differences were observed between control and ASA or MeSa treated fruit. Respiration rate decreased sharply during storage from day 0 to day 30 and then more slowly after that, and it was lower in SA treated fruits than in controls during the whole storage time (Supplementary Figure S1B).

**FIGURE 3:**
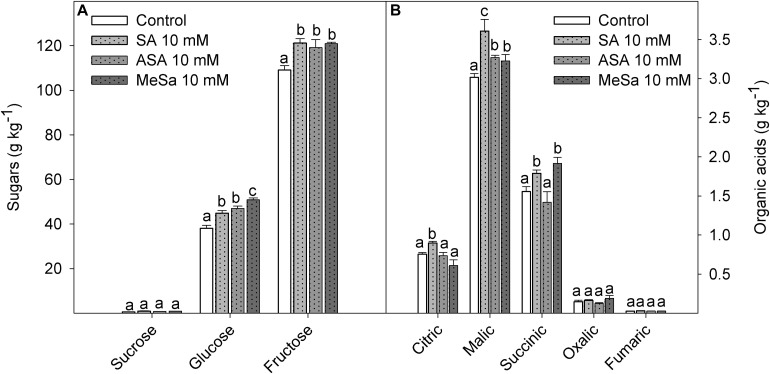
Concentration of major individual sugars **(A)** and organic acids **(B)** in arils of pomegranate from control and salicylic acid (SA), acetyl salicylic acid (ASA), and methyl salicylate (MeSa) treated fruits at harvest, in the 2018 experiment. Data are the mean ± SE. Different letters show significant differences (*P* < 0.05) among treatments for each individual sugar and organic acid.

Fruit firmness at harvest was significantly (*P* < 0.05) increased by SA and ASA treatments, from values of 15.89 ± 0.74 kN m^–1^ in control fruit to 18.46 ± 0.46 and 18.88 ± 0.74 kN m^–1^ in those treated with SA and ASA, respectively, confirming the results obtained in the 2017 experiment. During storage, fruit firmness decreased in control and treated fruits, although final firmness levels after 90 days of storage at 10°C remained significantly higher (*P* < 0.05) in fruits from treated trees than in controls (Supplementary Figure S2A). With respect to arils color, lower values of Hue angle were measured at harvest in fruits from SA, ASA, and MeSa treated trees. Hue angle of arils decreased during storage, although values of control fruits were always higher than those of treated ones (Supplementary Figure S2B). These results indicated that the aril red color increased as a consequence of salicylate treatments, either at harvest or during storage for 90 days, as can be observed in Supplementary Figure S3. Fructose was the major sugar in pomegranate arils, followed by glucose, and both sugars were significantly (*P* < 0.05) increased by salicylate treatments, while sucrose was found at a very low concentration without significant differences attributed to treatments (Figure 3A). Organic acids were also increased by salicylate treatments, the highest increase being found in the major organic acid, malic acid, by SA treatment (Figure 3B).

### Bioactive Compounds and Antioxidant Activity During Storage

**FIGURE 4:**
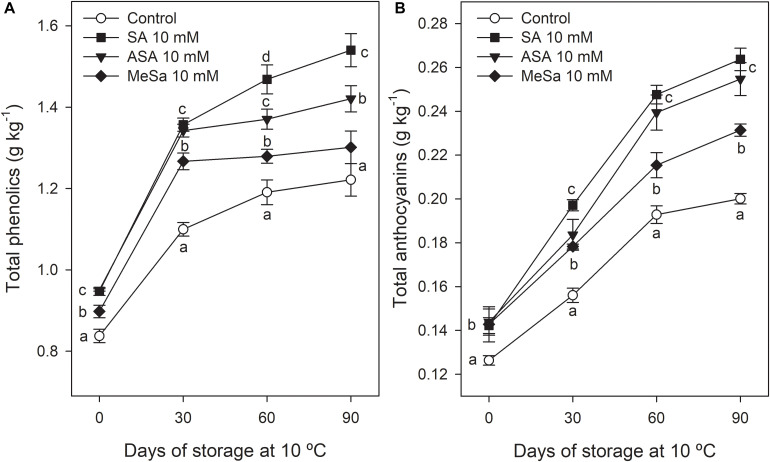
Total phenolic **(A)** and anthocyanin **(B)** concentration in arils of pomegranate from control and salicylic acid (SA), acetyl salicylic acid (ASA), and methyl salicylate (MeSa) treated fruits during storage at 10°C, in the 2018 experiment. Data are the mean ± SE. Different letters show significant differences (*P* < 0.05) among treatments for each sampling date.

**FIGURE 5:**
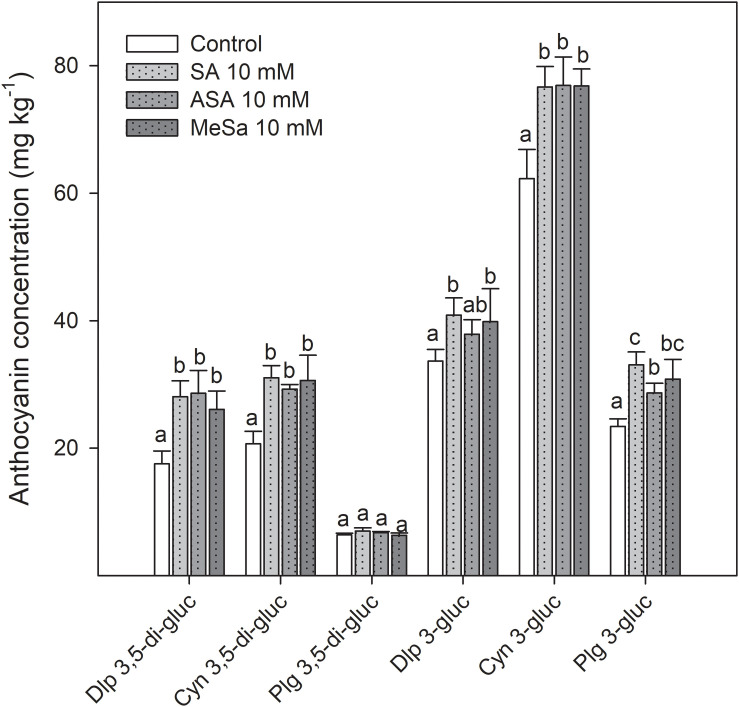
Concentration of delphinidin 3,5-di-*O*-glucoside (Dlp 3,5-di-gluc), cyanidin 3,5-di-*O*-glucoside (Cyn 3,5-di-gluc), pelargonidin 3,5-di-*O*-glucoside (Plg 3,5-di-gluc), delphinidin 3-*O*-glucoside (Dlp 3-gluc), cyanidin 3-*O*-glucoside (Cyn 3-gluc), and pelargonidin 3-*O*-glucoside (Plg 3-gluc) in arils of pomegranate from control and salicylic acid (SA), acetyl salicylic acid (ASA), and methyl salicylate (MeSa) treated fruits at harvest, in the 2018 experiment. Data are the mean ± SE. Different letters show significant differences (*P* < 0.05) among treatments for each individual anthocyanin.

In the 2018 experiment, SA, ASA, and MeSa treatments at 10 mM led to arils with increased concentrations of total phenolics and total anthocyanins at harvest time (Figures 4A,B), in agreement with results from the 2017 experiment. Total phenolic concentration increased during storage in pomegranate arils for all treatments, the major increases being found during the first month of storage. At the last sampling date, phenolic concentration was 1.22 ± 0.04 g kg^–1^ in arils from control fruit, and significantly higher (*P* < 0.05), in those from SA, ASA, and MeSa treated ones, at 1.54 ± 0.04, 1.42 ± 0.03, and 1.30 ± 0.04 g kg^–1^, respectively (Figure 4A). For total anthocyanin concentration, values at harvest were significantly higher (*P* < 0.05) in arils from treated fruits than in those from controls, without significant differences among SA, ASA, or MeSa treatments. Anthocyanin concentration also increased during the whole storage period and was higher in arils from all treated fruits than in controls, the highest increase being found in arils of SA and ASA treated fruits (Figure 4B). Individual anthocyanin concentration was measured at day 0 in the arils from control and treated fruits. In all aril samples a similar anthocyanin profile was found, Cyn 3-gluc being the major anthocyanin (62.29 ± 4.58 mg kg^–1^ in control fruit), followed by Dlp 3-gluc, Plg 3-gluc, Cyn 3,5-di-gluc, and Dlp 3,5-di-gluc while Plg 3,5-di-gluc was found at a very low concentration (Figure 5). It is worth noting that all individual anthocyanins, except those found at very low concentrations, were increased as a consequence of salicylate treatments, although no significant differences were observed between SA, ASA, and MeSa treatments.

**FIGURE 6:**
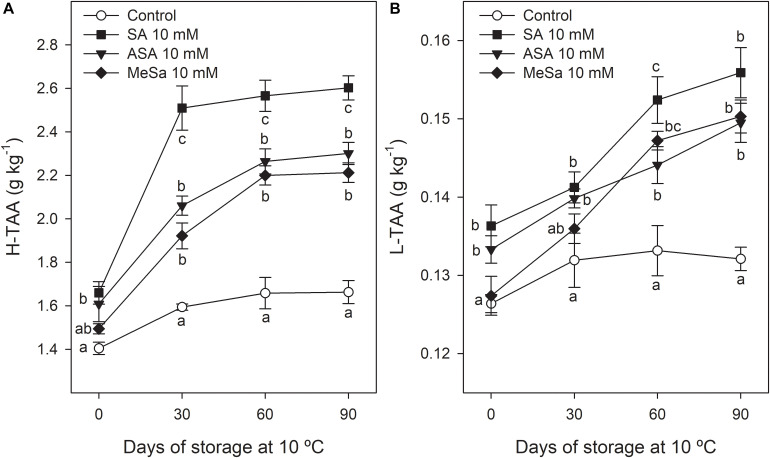
Total hydrophilic antioxidant activity (H-TAA) **(A)** and total lipophilic antioxidant activity (L-TAA) **(B)** in arils of pomegranate from control and salicylic acid (SA), acetyl salicylic acid (ASA), and methyl salicylate (MeSa) treated fruits during storage at 10°C, in the 2018 experiment. Data are the mean ± SE. Different letters show significant differences (*P* < 0.05) among treatments for each sampling date.

Similar trends than those for phenolic and anthocyanin content was observed for H-TAA in the arils, which increased during storage, especially in fruits from SA treated trees, while similar increases were found in arils from ASA and MeSa treatments, although they were significantly higher than in arils from control fruits (Figure 6A). However, L-TAA showed very low values as compared with H-TAA, its increase during storage was small and differences between control and treated fruits were only significant at the last two sampling dates (Figure 6B).

**FIGURE 7:**
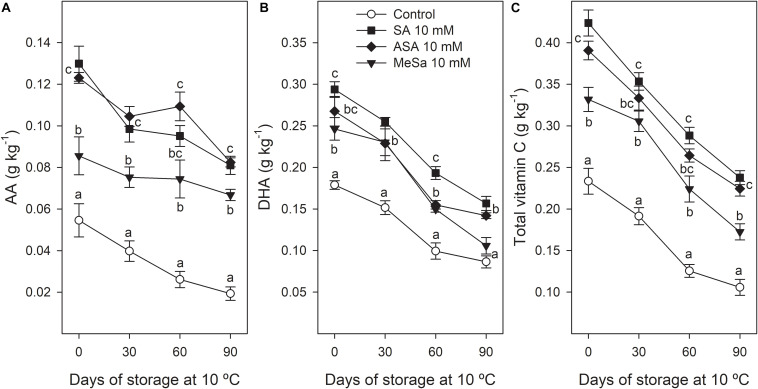
Ascorbic acid (AA) **(A)**, dehydroascorbic acid (DHA) **(B)**, and vitamin C **(C)** in arils of pomegranate from control and salicylic acid (SA), acetyl salicylic acid (ASA), and methyl salicylate (MeSa) treated fruits during storage at 10°C, in the 2018 experiment. Data are the mean ± SE. Different letters show significant differences (*P* < 0.05) among treatments for each sampling date.

Ascorbic acid content was significantly (*P* < 0.05) increased due to salicylate treatments, with values at harvest of 0.055 ± 0.008 g kg^–1^ in arils of control fruit, 0.086 ± 0.009 g kg^–1^ in those treated with MeSa, and 0.130 ± 0.008 and 0.123 ± 0.003 g kg^–1^ in arils of SA and ASA treated fruits. During storage, AA acid concentration decreased in all treatments, although higher values were found in arils of all treated fruits than in controls during the whole storage time, with final values of 0.019 ± 0.003 g kg^–1^ in control fruit and between 0.067 and 0.082 g kg^–1^ in treated ones (Figure 7A). DHA concentration at harvest was also significantly increased (*P* < 0.05) by salicylate treatments and maintained at higher levels during storage, although the effects were not as pronounced as in ascorbic acid (Figure 7B). Total vitamin C, calculated as the sum of AA plus DHA, followed a similar trend, with significantly higher values (*P* < 0.05) in treated than in control fruits, at harvest and along the whole storage time (Figure 7C).

## Discussion

Crop management, and especially water availability, during pomegranate fruit development has been reported to affect fruit ripening, quality, and phytochemical content (Galindo et al., 2014). In the present experiments, climatic conditions were similar for 2017 and 2018 and control and salicylate treated trees were under similar climatic and agronomic conditions. Thus, differences among control and treated trees on yield, fruit ripening, and quality attributes would be due just to the effects of treatments. Results showed that SA, ASA, and MeSa treatments increased crop yield due to an increase in the number of fruits that were harvested from each tree, which had a similar mass, independently of the applied treatment. The increase in fruit number by salicylate treatments could be due to: (i) an increased flowering rate, (ii) an increased rate of set fruits, or (iii) a decrease in fruit abscission. However, in our experiments, treatments were performed when fruit had reached ca 30% of their final size so that flowering or fruit set were not affected and the increase in fruit number was due to the reduction of fruit abscission that naturally occurs during the fruit developmental process. Accordingly, an increase in plum tree yield has been recently reported as a consequence of salicylate treatments, although it was due to increased fruit mass but not fruit number (Martínez-Esplá et al., 2018). Moreover, significant increases in total yield and cluster weight of ‘Flame Seedless’ table grapes were observed as a consequence of vine treatment with a foliar spray of 1.5 or 2.0 mM SA (Champa et al., 2015). These results prove that treatment with salicylates increases net photo-assimilate production in plants and/or the sink strength of developing fruits. In fact, foliar application of SA to ginger plants increased leaves’ chlorophyll content, photosynthetic rate, and total dry weight (Ghasemzadeh and Jaafar, 2013). Moreover, an increase in Rubisco activity and total yield by SA treatment were also reported in maize and mustard plants (Fariduddin et al., 2003; Elgamaal and Maswada, 2013). However, the recent results also show an effect on reducing the abscission of pomegranate fruit. Accordingly, Helaly et al. (2018) found a higher percentage of fruit retention and crop yield on two mango cultivars for three consecutive years as a consequence of SA treatments. These effects were attributed to the role of SA activating growth and the nutritional state of trees due to an increase in fresh and dry weight and chlorophyll, carotenoid, and sugar concentration in leaves, showing an effect on enhancing net photosynthesis on tree. In addition, given the pivotal role of SA on increasing tree tolerance to environmental stresses (Tiwari et al., 2017; Koo et al., 2020), its effect allowing pomegranate tress to overcome drought and high temperature stresses during summer seasons in the Spain Southeast cannot be discarded. In fact, maximum mean temperatures in summer, from June to September, were very high in the field crop, ca. 31.5°C in both years. On the other hand, it is worth noting that the percentage of fruits harvested in the first picking date was higher in 5 and 10 mM treated trees and in all ASA treated ones than in controls (Table 1). These results show that the on-tree ripening process was accelerated by SA and ASA treatments, especially when applied at 10 mM concentration since all trees (control and treated) were located on the same farm and under similar agronomic and environmental conditions, and pomegranate fruits were harvested upon reaching their commercial ripening stage, according to their size and skin color. This effect could be attributed to an increase of net photosynthesis and/or sink strength induced by treatment with salicylates previously commented (Fariduddin et al., 2003; Elgamaal and Maswada, 2013; Ghasemzadeh and Jaafar, 2013). This is important to point out the rule showing an inverse relationship between tree fruit load and fruit quality. In this experiment, despite that, the number of fruits was higher in treated trees but the fruit quality was not decreased.

Fruit firmness, TA, TSS, and skin and aril color are indicators of pomegranate fruit quality (Nuncio-Jáuregui et al., 2014; Valero et al., 2014; Pareek et al., 2015). Then, the higher firmness, TSS, and TA and the lower skin and aril Hue angle values found at harvest in the pomegranates of treated trees show that they had higher quality attributes than controls. It is worth noting that the major sugars, fructose and glucose, and the major organic acid, malic acid, were found at higher concentrations in arils of treated fruit than in control (Figure 3A). This sugar profile is in agreement with previous reports on ‘Mollar de Elche’ and other sweet pomegranate cultivars, while in sour cultivars the major organic acid is citric acid (Nuncio-Jáuregui et al., 2014; Cano-Lamadrid et al., 2018). In addition, increases in weight losses (Supplementary Figure S1) and decreases in firmness (Supplementary Figure S2A) and arils Hue angle (Supplementary Figure S2B) show the normal evolution of the postharvest ripening process in “Mollar de Elche” and other pomegranate cultivars (Sayyari et al., 2011a, 2016; García-Pastor et al., 2020), which were delayed by pre-harvest salicylate treatments. Accordingly, the evolution of the postharvest ripening process was delayed in two sweet cherry cultivars by pre-harvest treatments with SA, ASA, and MeSa leading to maintenance of fruit quality parameters (Valverde et al., 2015; Giménez et al., 2017). In this non-climacteric fruit species, as well as in plum (Martínez-Esplá et al., 2017), which is a climacteric fruit, the effect of salicylate preharvest treatments on delaying the postharvest ripening process was attributed to an increase on the concentration of antioxidant compounds and the activity of the antioxidant enzymes. The enhance of these antioxidant systems could lead to efficient scavenging of reactive oxygen species (ROS) which are generated during fruit ripening, a process considered as a functionally modified protracted form of senescence (Hodges et al., 2004).

Pomegranate fruits are a rich source of bioactive compounds, such as phenolics, including anthocyanins and other complex flavonoids and hydrolyzable tannins, and ascorbic acid, as compared with other fruits of the Mediterranean diet, although they are found at different concentrations depending on cultivar, cultural practices, and environmental conditions (Mphahlele et al., 2014; Li et al., 2015; Attanayake et al., 2018). These bioactive compounds have antioxidant properties which are responsible for the beneficial health effects attributed to pomegranate fruit consumption (Faria and Calhau, 2011; Asgary et al., 2017; Panth et al., 2017). Results of the present research show that pre-harvest treatments with salicylates increased total phenolic compound and total and individual anthocyanin concentrations, as well as ascorbic acid, leading to increases in H-TAA. These effects of salicylate treatments on increasing antioxidant compounds were significant at harvest and were maintained during long term storage (Figures 2A,B,C, 4A,B, 5, 6A, 7A) showing that pre-harvest treatment with salicylates would provide the fruit with increased beneficial health effects for human consumption. In fact, it has been claimed that these hydrophilic compounds are responsible for the antioxidant properties of a wide range of fresh fruit species (Valero and Serrano, 2010; Valverde et al., 2015; Martínez-Esplá et al., 2018). This statement is supported by the present results which show that H-TAA was 10-fold higher than L-TAA and strongly correlated *r*^2^ = 0.827 (*y* = 1.726x–0.120) and *r*^2^ = 0.753 (*y* = 7.929x + 0.439) with total phenolic and total anthocyanin content, respectively, by taking into account data of all treatment and sampling dates of the 2018 storage experiment.

On the other hand, it is worth noting that AA concentration in pomegranate arils decreased during storage, which is a general trend previously reported in other fruit species such as kiwifruit (Yang et al., 2016), peaches (Falagán et al., 2016) and even in “Hicaznar” (Koyuncu et al., 2019) and “Mridula” (Barman et al., 2014) pomegranate cultivars. However, AA concentration was higher on arils of pomegranates from treated trees than in controls, at harvest and along the storage process. Accordingly, postharvest SA treatment at 2 mM delayed the decrease in AA during storage at 2°C + 2 days at 20°C in “Malas Saveh” pomegranate (Sayyari et al., 2009), as well as in “Malase Yazd” pomegranate, especially if SA dipping treatment was applied as a hot solution (Dokhanieh et al., 2016). This effect could be attributed to an increase in the GR/APX system activity, to lower activity of ascorbic acid oxidase activity (AAO), and/or to higher reducing sugar (glucose and fructose) accumulation. It has been reported that AA is oxidized to DHA by AAO during storage, leading to increases in DHA in a wide range of fresh fruits (Mazurek and Pankiewicz, 2012), including kiwifruit (Yang et al., 2016). However, in the present experiment, DHA decreased in pomegranate arils during storage (Figure 7B), as has been reported to occur in peaches (Falagán et al., 2016). Nevertheless, DHA concentration was maintained at higher levels in salicylate treated fruits than in controls, leading to higher concentration in vitamin C (Figure 7C), which is an important effect of salicylate treatments on the nutritional value of pomegranate, along with the enhancing of its antioxidant properties.

No previous reports are available in the literature regarding the effect of pre-harvest salicylate treatments on increasing phenolic and anthocyanin content in pomegranate fruit, although some papers exist on other fruits for comparative purposes. In this sense, SA, ASA, and MeSA applied as pre-harvest treatments on sweet cherry and plum trees increased fruit total phenolic and anthocyanin concentrations at harvest with respect to controls, and these differences were maintained during cold storage (Giménez et al., 2014, 2015, 2017; Martínez-Esplá et al., 2018). Accordingly, SA pre-harvest treatments of vine led to higher levels of these bioactive compounds in table grape “Flame Seedless” at harvest and during postharvest storage (Champa et al., 2015). On pomegranate fruits, postharvest treatments with SA, ASA, or MeSA have been reported to maintain total phenolics, anthocyanins, and antioxidant activity at higher levels than in control fruit during cold storage (Sayyari et al., 2011a, b) as well as SA and ASA on sweet cherry (Valero et al., 2011), and SA on cornelian cherry fruit (Dokhanieh et al., 2013) and apricot (Wang et al., 2015). These enhancements were attributed to an increase in the activity of phenylalanine ammonia lyase (PAL), which is the main enzyme involved in the biosynthetic phenolic pathway. Accordingly, dipping of pomegranate arils with SA maintained higher anthocyanin and phenolic concentrations during storage, the effect being higher if SA dipping was performed at 45 than at 25°C and also attributed to the enhanced activity of PAL (Dokhanieh et al., 2016).

## Conclusion

Overall results show that, in the 2017 and 2018 experiments, salicylate pre-harvest treatments of pomegranate trees increased crop yield (kg tree^–1^ and number of harvested fruit tree^–1^) and fruit quality parameters at harvest, such as firmness, aril color, and individual sugar and organic acid contents. Moreover, aril content on bioactive compounds, such as phenolics, anthocyanins, and ascorbic acid, was also increased by salicylate treatments. The quality traits and the concentration of bioactive compounds were maintained at higher levels in pomegranate fruit from treated trees than in controls during prolonged storage at 10°C. It is worth noting the effects of these treatments on increasing total and individual anthocyanin concentration in pomegranate arils which reached a deeper red color and, in turn, would be more appreciated in the international market, all these effects contributing to increasing the profit of this crop. Thus, pre-harvest treatment with salicylates, and especially SA at 10 mM, could be a safe and natural new tool to improve pomegranate fruit quality and their content on antioxidant compounds with beneficial health effects, at harvest and during storage.

## Data Availability Statement

All datasets generated for this study are included in the article/Supplementary Material.

## Author Contributions

DV and MS conceived and designed the work in association with other authors. SC, PZ, and DM-R performed the field treatments. MG-P performed most of the analytical determination in collaboration with the other authors. MS and DV analyzed the data and wrote the manuscript. All authors approved the final version of the manuscript.

## Conflict of Interest

The authors declare that the research was conducted in the absence of any commercial or financial relationships that could be construed as a potential conflict of interest.

## Footnotes

1 http://riegos.ivia.es/datos-meteorologicos

## Supplementary Material

FIGURE S1 Weight loss **(A)** and respiration rate **(B)** in arils of pomegranate from control and salicylic acid (SA), acetyl salicylic acid (ASA), and methyl salicylate (MeSa) treated fruits during storage at 10°C, in the 2018 experiment. Data are the mean ± SE. Different letters show significant differences (*P* < 0.05) among treatments for each sampling date.

FIGURE S2 Fruit firmness **(A)** and aril color (Hue angle) **(B)** in pomegranate from control and salicylic acid (SA), acetyl salicylic acid (ASA), and methyl salicylate (MeSa) treated fruits during storage at 10°C, in the 2018 experiment. Data are the mean ± SE. Different letters show significant differences (*P* < 0.05) among treatments for each sampling date.

FIGURE S3 Pictures of the cut surface of pomegranate from control and salicylic acid (SA), acetyl salicylic acid (ASA), and methyl salicylate (MeSa) treated fruits at harvest **(A)** and after 90 days of storage at 10°C **(B)**, in the 2018 experiment. Pictures represent one of the three replicates for each treatment.
