# Supplementary figures and images for: The Effects of Salicylic Acid and Its Derivatives on Increasing Pomegranate Fruit Quality and Bioactive Compounds at Harvest and During Storage
Source: Front Plant Sci. 2020 Jul 1;11:668. doi: 10.3389/fpls.2020.00668 (PMC7344906; doi:10.3389/fpls.2020.00668)

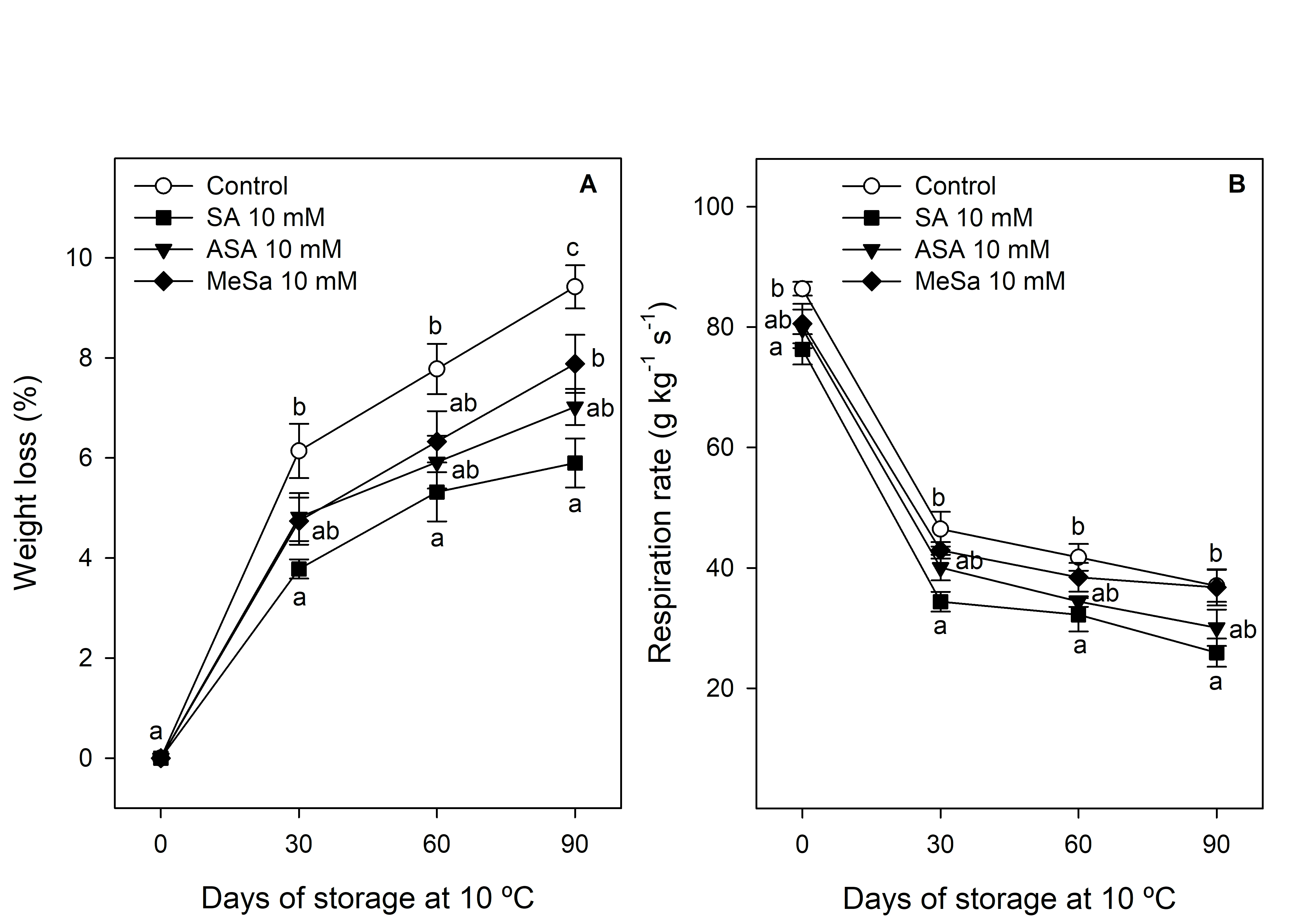

Supplement: FIGURE S1 — Weight loss (A) and respiration rate (B) in arils of pomegranate from control and salicylic acid (SA), acetyl salicylic acid (ASA), and methyl salicylate (MeSa) treated fruits during storage at 10°C, in the 2018 experiment. Data are the mean ± SE. Different letters show significant differences (P < 0.05) among treatments for each sampling date. [file Image_1.TIF]

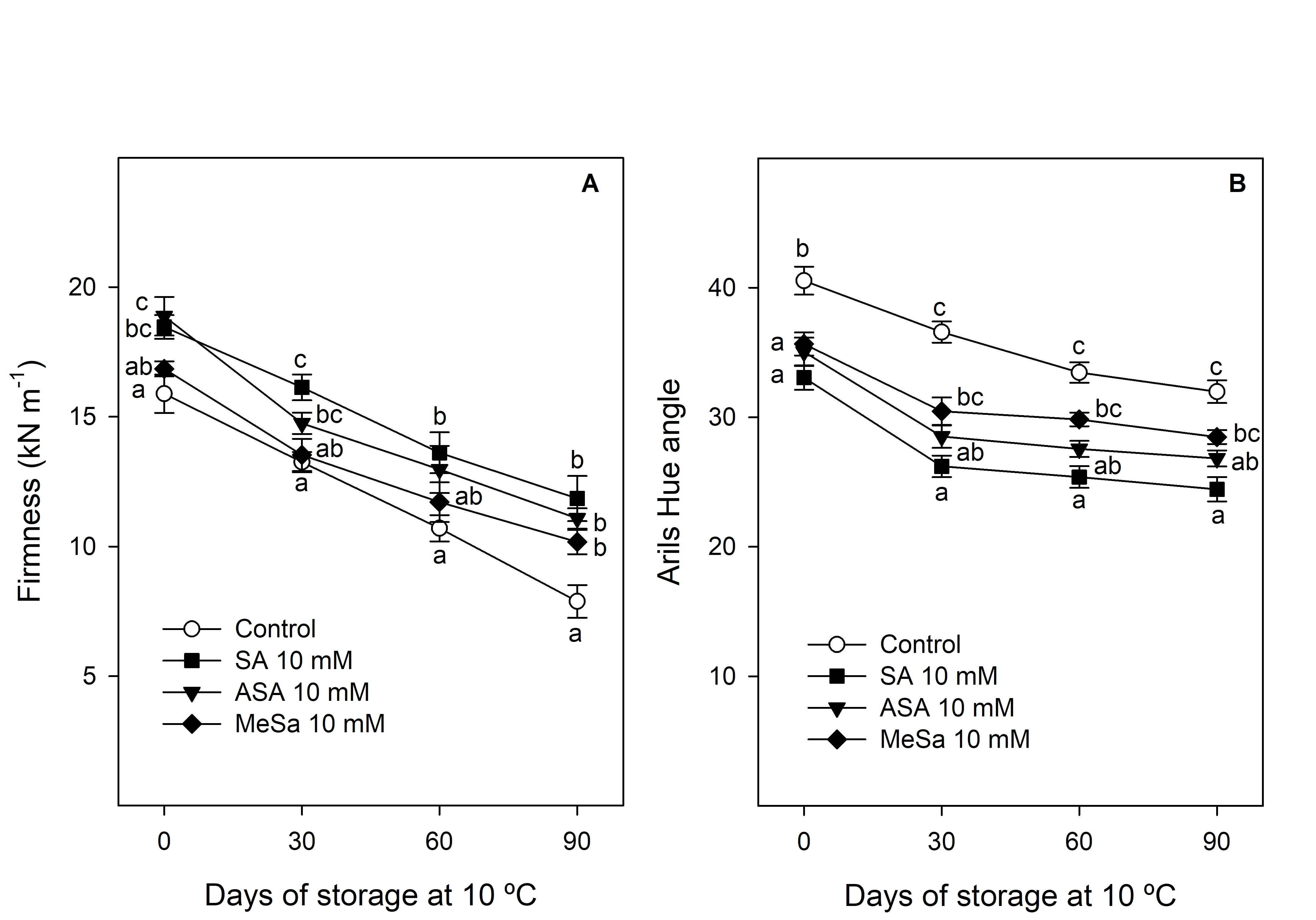

Supplement: FIGURE S2 — Fruit firmness (A) and aril color (Hue angle) (B) in pomegranate from control and salicylic acid (SA), acetyl salicylic acid (ASA), and methyl salicylate (MeSa) treated fruits during storage at 10°C, in the 2018 experiment. Data are the mean ± SE. Different letters show significant differences (P < 0.05) among treatments for each sampling date. [file Image_2.TIF]

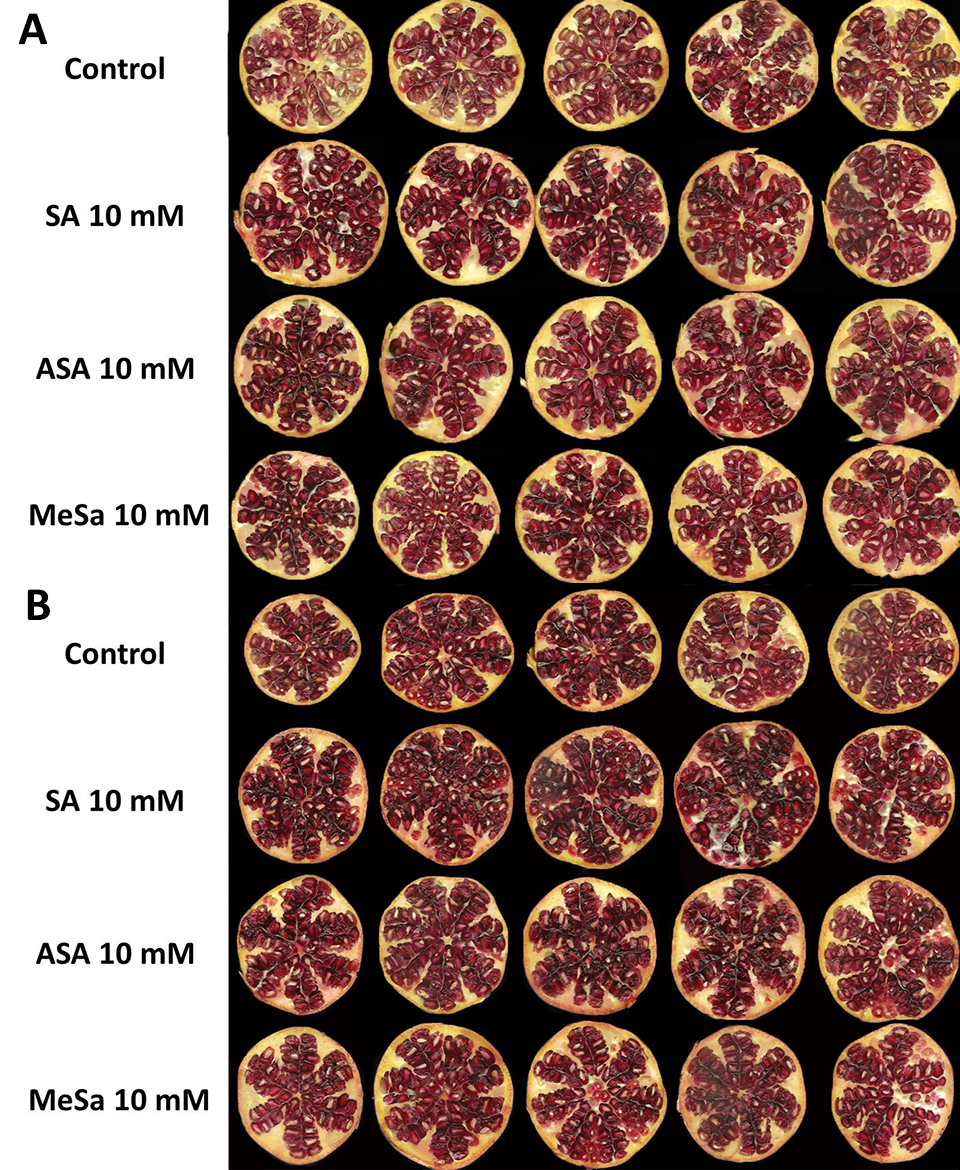

Supplement: FIGURE S3 — Pictures of the cut surface of pomegranate from control and salicylic acid (SA), acetyl salicylic acid (ASA), and methyl salicylate (MeSa) treated fruits at harvest (A) and after 90 days of storage at 10°C (B), in the 2018 experiment. Pictures represent one of the three replicates for each treatment. [file Image_3.TIF]
